# Acute, Severe Hepatitis of Unknown Origin: Should We Really Be Afraid of Another Obscure Enemy of Our Children?

**DOI:** 10.3390/pediatric14020029

**Published:** 2022-05-03

**Authors:** Maurizio Aricò, Désirée Caselli

**Affiliations:** 1 Pediatrics, Ospedale S. Spirito, Azienda Sanitaria Locale, 65121 Pescara, Italy; 2 Pediatric Infectious Diseases, Giovanni XXIII Children Hospital, Azienda Ospedaliero Universitaria Consorziale Policlinico, 70121 Bari, Italy; desiree.caselli@policlinico.ba.it

On 31 March 2022, Public Health Scotland was alerted to five children aged 3–5 years, presenting to the Glasgow children’s hospital with severe hepatitis of unknown etiology within a 3-week period. This cluster obviously exceeded the expected number of cases of hepatitis of unknown etiology, fewer than four per year [1]. A multi-disciplinary expert team reviewed clinical and epidemiological data from the first five children: vomiting in preceding weeks, jaundice, and exceptionally high levels of transaminases, often greater than 2000 international units per liter (IU/L; n.v. <40 IU/L) were the main features [2]. 

As of 5 April 2022, WHO was informed on 10 Scottish cases of severe acute hepatitis with unknown etiology in children aged < 10 years from Scotland. Nine presented during March 2022, one during the previous January. The clinical features were jaundice, diarrhea, vomiting, and abdominal pain. All ten cases were retrieved from hospital records. One child underwent liver transplant. 

As of 12 April 2022, 74 cases had been reported in the United Kingdom since January 2022, 49 in England. Of the 13 from Scotland, three cases were SARS-CoV-2 positive, five were negative, and two had evidence of recent, previous infection. Eleven of the 13 were tested for Adenovirus, with five positive. 

As of 21 April 2022, at least 169 cases of acute hepatitis of unknown origin have been reported from 11 countries in the WHO European Region and one country in the WHO Region of the Americas. Cases have been reported in the United Kingdom of Great Britain and Northern Ireland (*n* = 114), Spain (*n* = 13), Israel (*n* = 12), the United States of America (*n* = 9), Denmark (*n* = 6), Ireland (<5), the Netherlands (*n* = 4), Italy (*n* = 4), Norway (*n* = 2), France (*n* = 2), Romania (*n* = 1), and Belgium (*n* = 1). Cases are aged 1 month to 16 years old. Seventeen children (approximately 10%) have required liver transplantation; at least one death has been reported [3]. 

## 1. WHO Working Case Definition 

**Confirmed:** N/A at present

**Probable:** A person presenting with an acute hepatitis (**non hepA-E** (If hepatitis A-E serology results are awaited, but other criteria met, these can be reported and will be classified as “pending classification”. Cases with other explanations for their clinical presentation are discarded)) with serum transaminase >500 IU/L (AST or ALT), who is 16 years and younger, since 1 October 2021.

**Epi-linked**: A person presenting with an acute hepatitis (**non hepA-E** (If hepatitis A-E serology results are awaited, but other criteria met, these can be reported and will be classified as “pending classification”. Cases with other explanations for their clinical presentation are discarded)) of any age who is a close contact of a probable case, since 1 October 2021. 

## 2. What Have We Learned, So Far?

This new story started a couple of weeks ago, while we are still struggling with a large number of children with variable clinical conditions, who—often unexpectedly—test positive for SARS-CoV-2. Now, we have to add another variable to our working plan. 

Based on numbers of reported cases, as summarized above, we might be induced to think that this is not going to be a big issue, or at least not an issue of big numbers. However, obviously, the COVID-19 lesson is still on the scene, and we must take every caution before running the risk of under evaluating this new scenario.

Every single child requiring “unexpected” (based on epidemiology of the last years) liver transplant is a major issue for this child, the family and public health. 

Are we facing a new event? Although the hypothesis of a toxic origin cannot yet be ruled out, the working hypothesis in the scientific community is that this is an infectious, viral hepatitis. By definition, common hepatotropic viruses are excluded in these cases. Is SARS-CoV-2 playing a role? The first picture suggest this is not the case, since association between the two events is not convincing, with many children not infected by SARS-CoV-2 by the time acute hepatitis is reported. SARS-CoV-2 was identified in 20 cases of those that were tested. Furthermore, 19 were detected with a SARS-CoV-2 and adenovirus co-infection.

## 3. Adenovirus: Is He Guilty?

Another player is on the scene: Adenovirus has been detected in at least 74 cases. Is hepatitis expected in children with newly diagnosed Adenovirus infection?

Adenoviruses typically spread from person-to-person and most commonly cause respiratory illness, but depending on the type, can also cause other illnesses such as gastroenteritis, conjunctivitis, and cystitis. 

Whether clinical heterogeneity depends on the over 100 genotypes and 52 serotypes of Adenovirus, grouped in seven species designated Adenovirus-A through -G, remains undefined. Different types display different tissue tropisms, which may correlate with clinical manifestation. Moreover, they may circulate at a given time in different countries or regions. Transmission of novel strains between countries or across continents and replacement of dominant viruses by new strains may thus occur. 

Adenovirus type 41, the apparently implicated adenovirus type, typically presents as diarrhea, vomiting, and fever, often accompanied by respiratory symptoms. While there have been case reports of hepatitis in immunocompromised children with adenovirus infection, adenovirus type 41 is not known to be a cause of hepatitis in otherwise healthy children [3,4].

What is the role of Adenovirus in the landscape of viral hepatitis in children? In a recent systematic review of the global epidemiology of viral-induced acute liver failure, the burden of acute liver failure after infection with hepatitis B virus, hepatitis A virus, hepatitis C virus, hepatitis E virus, herpes simplex virus/human herpesvirus, cytomegalovirus, Epstein-Barr virus and parvovirus B19 was estimated. The prevalence of hepatitis A-induced acute liver failure was markedly lower in countries with routine hepatitis A immunization versus no routine hepatitis A immunization. Hepatitis E virus was the most common etiological cause of viral-induced acute liver failure reported in this review. In addition, viral-induced acute liver failure had poor outcomes as indicated by high fatality rates, which appear to increase with poor economic status of the studied countries.

Unfortunately, data were largely missing for acute liver failure after infection with varicella-zoster virus, human parainfluenza viruses, yellow fever virus, coxsackievirus and/or Adenovirus [5]. 

## 4. Open Questions

What is the spontaneous course of this new “*acute, severe hepatitis of unknown origin in children*”? It has been reported that a small minority of children with acute onset hepatitis and defective hepatocellular function, rapidly progress to end-stage liver failure, thus requiring urgent liver transplant. What about the remaining cases? There is evidence of retrospective diagnosis in a relatively large proportion of cases, with presumably favorable course, even in the absence of specific treatment, other than supportive therapy. Is this clinical course monophasic? Thus, we can reliably say that when a child with levels of transaminases exceeding 500 IU/L has shown a reduction to normal or almost normal levels the episode is likely closed? Or do we rather need to monitor this child to rule out that possible fluctuation still might herald a second spike and a possible clinical picture of hepatitis or even liver failure?

Furthermore, is there a correlation between the detectable number of Adenovirus copies and clinical course? Sequencing of the Adenovirus isolates from children with acute, severe hepatitis of unknown origin in children has the potential to offer a novel insight into this picture and clarify the possible role of type 41. To this issue, US CDC is warning about the observed differences in adenovirus test sensitivity for different specimen types, with tests using whole blood in some cases being more sensitive than those using plasma [6].

Is this a real, new threat for our children? Time will tell.

## Data Availability

No data-base was generated.
